# Tirzepatide vs. semaglutide for obesity, glycemic control, and cardiovascular outcomes: a narrative review of clinical trials

**DOI:** 10.3389/fmed.2026.1764664

**Published:** 2026-04-22

**Authors:** Maan H. Harbi, Ahmad M. Ashour, Nasser M. Alorfi, Mohammed M. Aldurdunji, Saad M. Wali, Yahya A. Alzahrani

**Affiliations:** 1 Pharmacology and Toxicology Department, College of Pharmacy, Umm Al-Qura University, Makkah, Saudi Arabia; 2 Pharmaceutical Practices Department, College of Pharmacy, Umm Al-Qura University, Makkah, Saudi Arabia; 3 Department of Pharmacology, Faculty of Medicine, King Abdulaziz University, Rabigh, Saudi Arabia

**Keywords:** comparative effectiveness, glycemic control, narrative review, obesity pharmacotherapy, Semaglutide, tirzepatide, weight loss

## Abstract

**Background:**

Tirzepatide, a dual glucose-dependent insulinotropic polypeptide, (GIP) and glucagon-like peptide-1 (GLP-1) receptor agonist, has emerged as an effective therapy for obesity and type 2 diabetes mellitus (T2DM). Its dual-incretin mechanism may offer enhanced metabolic benefits compared with selective GLP-1 receptor agonists such as semaglutide.

**Methods:**

A structured narrative review of clinical trials, real-world observational studies, and contextual cardiovascular outcome analyses was conducted. Literature was sourced from ClinicalTrials.gov and relevant scientific databases to compare tirzepatide and semaglutide across weight, glycemic, cardiometabolic, and safety outcomes.

**Results:**

Across completed head-to-head randomized trials, tirzepatide consistently achieved greater reductions in body weight, and HbA1c than semaglutide in individuals with obesity or T2DM. Semaglutide, however, has the most mature evidence for cardiovascular risk reduction, as demonstrated in the SUSTAIN-6, PIONEER-6, and SELECT trials. The SURPASS-CVOT trial established cardiovascular non-inferiority for tirzepatide compared with dulaglutide, alongside improvements in cardiometabolic risk factors. Real-world studies reported heterogeneous cardiovascular outcomes.

**Conclusion:**

Tirzepatide demonstrates superior metabolic efficacy in direct comparative trials, whereas semaglutide currently has the strongest evidence for cardiovascular benefit. Treatment selection should be individualized based on clinical priorities and patient characteristics.

## Introduction

The increasing prevalence of type 2 diabetes mellitus (T2DM) and obesity highlights the urgent clinical need for pharmacotherapies with substantial weight loss and optimal glycemic control (1). Incretin-based therapies, specifically agonists of glucagon-like peptide-1 receptors (GLP-1 RAs) and glucose-dependent insulinotropic polypeptide (GIP) receptors, have demonstrated benefits for regulating appetite, improving glucose homeostasis, and reducing cardiovascular risk (2, 3).

Semaglutide is a GLP-1 RA that has shown significant clinical advantages in both diabetic and non-diabetic populations. Landmark trials such as SUSTAIN and STEP demonstrated significant reductions in HbA1c levels and body weight in T2DM and obesity (4, 5). Tirzepatide represents the next generation of incretin-based therapy and is considered a novel dual agonist of GLP-1 and GIP receptors. This unique mechanism of action has been shown to produce greater reductions in body weight and HbA1c compared with traditional GLP-1 RAs in trials involving individuals with obesity or T2DM. These findings have generated significant interest regarding the comparative effectiveness of tirzepatide and semaglutide across metabolic, cardiovascular, and patient-centered outcomes (6, 7).

Although direct head-to-head evidence robustly favors tirzepatide for weight loss and glycemic control, comparative cardiovascular outcomes currently rely on contextual data from independent cardiovascular outcome trials and real-world observational studies. As this evidence base rapidly expands, a contemporary synthesis is required to inform clinical decision-making. Consequently, this structured narrative review evaluates the comparative effects of tirzepatide vs. semaglutide on weight, glycemic control, cardiovascular outcomes, and safety profiles in adults with obesity or T2DM.

## Methodological framework and research design

### Search strategy and inclusion criteria

This study is explicitly designed as a structured narrative review. To ensure methodological clarity, avoid overstating analytical rigor and to address the inherent heterogeneity of comparing controlled clinical trials with observational real-world evidence, no original quantitative meta-analysis or formal risk-of-bias assessments were conducted. A study selection flow diagram is provided to enhance transparency of the literature identification and screening process. This approach maintains a transparent structure for identifying and synthesizing the literature without claiming the strict reproducibility of a systematic review.

**PICOS framework and eligibility criteria** to provide methodological clarity, the research scope and eligibility criteria were strictly defined using the PICOS framework:

- **Population:** adults with obesity or overweight (with or without diabetes) or adults with type 2 diabetes mellitus (T2DM).
- **Intervention:** tirzepatide.
- **Comparators:** semaglutide (for direct comparisons). Contextual comparators, such as bariatric surgery or dulaglutide, were also permitted under specific circumstances to provide broader clinical context. Specifically, the inclusion of the SURPASS-CVOT trial, which compares tirzepatide against dulaglutide rather than semaglutide, is explicitly justified as it provides vital contextual cardiovascular outcome data for tirzepatide in the absence of a completed head-to-head cardiovascular trial between the two primary agents.
- **Outcomes:** clinically relevant variables including absolute and percentage change in body weight, HbA1c reduction, cardiometabolic outcomes (e.g., major adverse cardiovascular events [MACE], hospitalizations), and adverse event incidence (e.g., gastrointestinal symptoms, hypoglycemia).
- **Study Designs:** randomized controlled trials (RCTs), open-label interventional trials, and observational real-world cohort studies.

Studies were excluded if they focused on investigational therapies other than semaglutide, compared tirzepatide to combination therapies (e.g., CagriSema) rather than semaglutide directly, or investigated non-clinical outcomes (e.g., gastric motility) without reporting weight or glycemic endpoints. Furthermore, to ensure the outcome synthesis relied only on analyzable data, ongoing studies without published primary results are noted in the summary tables solely to provide context regarding the future research landscape; they are excluded from the main comparative data synthesis.

### Data sources and search strategy

To identify relevant literature, searches were conducted using the ClinicalTrials.gov database and PubMed. The search strategy utilized the following primary terms: (tirzepatide OR Mounjaro OR Zepbound) AND (semaglutide OR Ozempic OR Wegovy). These keywords were combined with condition-specific terms and filters, including “obesity”, “overweight”, “weight loss”, “type 2 diabetes”, “glycemic control”, and “metabolic syndrome”. The search included studies with results available up to June 10, 2025, and was restricted to completed trial records and full-text articles available in English. Reference lists of relevant published reviews and clinical guidelines were also manually screened to capture additional real-world comparative cohorts.

### Data extraction and synthesis

From the eligible studies, detailed variables were extracted to facilitate a structured narrative comparison. Extracted data included trial identifiers (NCT number, phase, duration), baseline population characteristics (baseline BMI, diabetes status), specific intervention arms and dosages, primary and secondary metabolic outcomes, and safety parameters. The extracted outcomes were synthesized narratively to evaluate comparative clinical effectiveness across the respective patient populations.

## Results

### Study identification and selection

**Figure 1:**
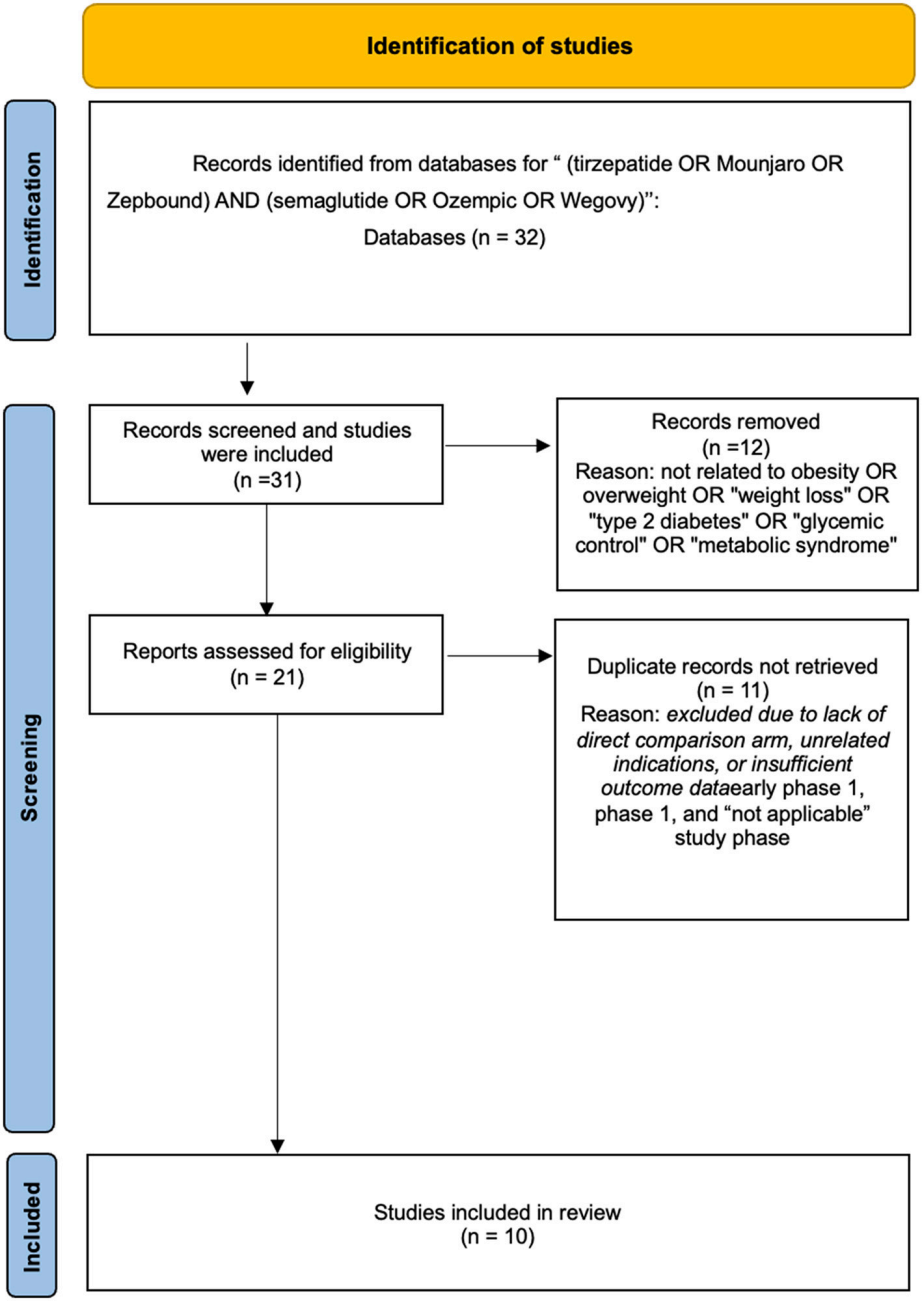
Study selection flow diagram for included studies (narrative review).

A comprehensive search of ClinicalTrials.gov and relevant databases was conducted to identify studies evaluating tirzepatide and semaglutide. Initially, 32 clinical study records were retrieved. After applying the defined PICOS inclusion and exclusion criteria to select studies directly comparing tirzepatide and semaglutide, a total of 10 studies were deemed eligible for this review. These consisted of five clinical trials, four real-world observational studies and the completed trial (SURPASS-CVOT) was included separately to serve as a contextual cardiovascular outcomes study. This reconciled study count aligns with the study selection process outlined in Figure 1.

### Characteristics of included studies

To ensure methodological clarity and appropriately weight the evidence, the included literature is organized into four distinct categories: completed head-to-head trials, contextual cardiovascular outcome trials, real-world observational studies, and ongoing (non-analytical) studies. Tables 1, 2 detail the study designs, populations, and primary outcomes of the included research.

1. Completed Head-to-Head Clinical Trials Two completed randomized controlled trials (SURPASS-2 and SURMOUNT-5) provided direct, head-to-head comparisons of tirzepatide and semaglutide. These trials evaluated populations with type 2 diabetes and obesity/overweight, respectively. They provide the primary analyzable clinical data for metabolic efficacy, specifically focusing on absolute and percentage changes in body weight and reductions in HbA1c (Table 3).
2. Contextual Cardiovascular Outcomes Trials (CVOTs) Currently, there are no completed head-to-head cardiovascular outcomes trials directly comparing tirzepatide and semaglutide. Therefore, the recently reported SURPASS-CVOT trial was included to provide vital contextual safety and efficacy data for tirzepatide. Involving over 13,000 adults with type 2 diabetes and established atherosclerotic cardiovascular disease, this trial demonstrated that tirzepatide was non-inferior to dulaglutide for three-point major adverse cardiovascular events (MACE). Furthermore, it achieved greater improvements in key cardiometabolic risk factors, including glycemic control, body weight, blood pressure, and lipid parameters (8) (Table 3).
3. Real-World Observational Studies Four real-world observational cohort studies were included to supplement the randomized clinical trial data. Utilizing large-scale electronic health records (EHR) and insurance claims databases (e.g., TriNetX), these studies evaluated the comparative effectiveness of tirzepatide vs. semaglutide in routine clinical practice. The studies featured follow-up periods ranging from 6 to 12 months and measured outcomes including on-treatment weight loss, HbA1c changes, and heterogeneous cardiovascular events (e.g., all-cause mortality, myocardial infarction, and stroke). The diverse demographics in these cohorts provide a robust foundation for comparing the agents outside of strictly controlled clinical environments.
4. Ongoing Studies (Non-Analytical) Three additional ongoing studies (two interventional and one observational) were identified that compare tirzepatide with semaglutide or bariatric surgery. Because these studies have not yet reached their primary completion dates (ranging from 2025 to 2027) and lack published results, they do not contribute analyzable outcome data to this review. They are clearly labeled as active or recruiting and are included in Table 1 strictly to map the future research landscape, particularly regarding heart failure with preserved ejection fraction (HFpEF) and surgical comparators.

**Table 1:** Summary of extracted clinical trials characteristics. (Updated from ClinicalTrials.gov on 10 June 2025).

NCT ID	Title	Status/design	Condition(s)	Intervention(s)	Primary outcome(s)	Start date	Primary completion	Locations
NCT06980623	Comparative effectiveness of Tirzepatide vs. Semaglutide in participants with type 2 diabetes and heart failure with preserved ejection fraction	Active not recruiting/interventional; 48-month follow-up	Type 2 diabetes|heart failure	Tirzepatide Semaglutide	Composite CV outcome, composite cardiovascular outcome includes hospitalization for HF or all-cause mortality, From treatment initiation to end of follow up, up to 48 months	1/1/25	6/1/25	United States
NCT06914141	Comparative effectiveness of Tirzepatide vs. Semaglutide in individuals with heart failure with preserved ejection fraction (DUP-TIRZSEMA)	Active not recruiting/observational	Diabetes Mellitus, type 2|HFpEF—heart failure with preserved ejection fraction	Tirzepatide Semaglutide	Composite of all-cause mortality or heart failure hospitalization, to evaluate the comparative effect of tirzepatide vs. semaglutide on all-cause mortality or heart failure hospitalization in patients with heart failure with preserved ejection fraction., Through study completion (1 day after cohort entry date until the first of outcome or censoring)	1/14/25	2025–05	United States
NCT06803888	Bariatric Surgery vs. Semaglutide vs. Tirzepatide	recruiting Randomized, parallel-group	Obesity and obesity-related medical conditions	PROCEDURE: bariatric surgery Semaglutide| Tirzepatide	The mean percentage weight loss, The mean percentage weight loss at 52 weeks for the following 2 comparisons: ^*^Bariatric surgery (RYGB and SG) vs. tirzepatide ^*^Bariatric surgery (RYGB and SG) vs. semaglutide, first 52 weeks of the study	1/29/25	7/1/27	United States
NCT03987919	A Study of Tirzepatide (LY3298176) vs. Semaglutide once weekly as add-on therapy to metformin in participants with type 2 diabetes **(SURPASS-2)**	Completed phase 3 RCT	Type 2 diabetes	Tirzepatide 5, 10, 15 mg vs. Semaglutide 1 mg weekly	Change From Baseline in Hemoglobin A1c (HbA1c) (10 mg and 15 mg), HbA1c is the glycosylated fraction of hemoglobin A. HbA1c is measured primarily to identify average plasma glucose concentration over prolonged periods of time. Least Squares (LS) mean was determined by mixed-model repeated measures (MMRM) model with Baseline + Pooled Country + Treatment + Time + Treatment\^*^Time (Type III sum of squares)., Baseline, Week 40	7/30/19	1/28/21	United States |Argentina Australia| Brazil| Canada| Israel| Mexico| Puerto Rico| United Kingdom
NCT05822830	A Study of Tirzepatide (LY3298176) in participants with obesity or overweight with weight related comorbidities (SURMOUNT-5)	Completed phase 3 RCT	obesity Overweight	Tirzepatide 10 & 15 mg vs. Semaglutide up to 2.4 mg weekly Semaglutide	Percent change from baseline in body weight, baseline, week 72	4/21/23	11/13/24	United States| Puerto Rico

**Table 2:** Key characteristics and findings of the major observational studies that evaluated tirzepatide vs. Semaglutide (or GLP-1 agonists) in real-world settings.

Study first author, year (publisher)	Data source & population	Follow-up	Outcomes measured	Key findings (tirzepatide vs. Semaglutide)
Rodriguez et al., 2024 (JAMA Intern Med) (30)	US EHR network; adults with obesity or T2DM	6–12 months	Weight, BMI, HbA1c	Tirzepatide produced significantly greater weight loss vs. semaglutide; limitations: on-treatment weight only
Terrell et al., 2025 (ISPOR poster) (26)	US claims data; T2DM and obesity	12 months post-initiation (required continuous insurance enrollment).	HbA1c change, Weight change (subsets with lab/weight data)	Greater weight loss and higher persistence with tirzepatide; industry-sponsored
Chuang et al., 2024 (JAMA Net Open) (31)	TriNetX; US cardiac-metabolic population	Median 10.5 months (IQR 5.2–15.7).	All-cause mortality; MACE (MI, stroke); MACE + mortality; Kidney outcomes (AKI, new kidney disease); HbA1c, weight change	Small CV differences between groups; tirzepatide had larger weight reductions
Dani et al., 2025 (JACC: Advances) (14)	TriNetX subset; high CV-risk adults	12 months	Composite CV outcome (MI, ischemic stroke, all-cause death); individual MACE components; safety outcomes not detailed	Lower MI, stroke, mortality with tirzepatide Limitation: small tirzepatide sample (*n* < 800) limits power; GLP-1 RA comparator includes older agents; confounding by indication possible even with matching (tirzepatide users might differ in unmeasured ways)

**Table 3:** Comparative outcomes between tirzepatide and Semaglutide in key trials.

Outcome	Tirzepatide	Semaglutide	Significance/notes
HbA1c reduction (SURPASS-2)	−2.01 to −2.30%	−1.86%	Favoring tirzepatide (*p* < 0.001)
Weight loss (kg) (SURPASS-2)	−7.6 to −11.2 kg	−5.7 kg	Favoring tirzepatide (*p* < 0.001)
Weight loss % (SURMOUNT-5)	−20.2%	−13.7%	Favoring tirzepatide
≥20% weight Loss Responders	50%	27%	Favoring tirzepatide
≥25% weight loss responders	33%	16%	Favoring tirzepatide
GI adverse events	17%−22%	~18%	Similar
Hypoglycemia	0.2%−1.7%	0.4%	Slightly higher with tirzepatide
Primary CV outcome: 3-point MACE (CV Death, MI, Stroke)	**SURPASS-CVOT:** non-inferior to dulaglutide; HR ~0.90–0.95 (directionally favoring tirzepatide but *not* meeting superiority)	**SELECT (no diabetes):** 20% MACE reduction; **SUSTAIN-6/PIONEER-6:** significant CV benefit in T2DM	Semaglutide has more mature randomized evidence for cardiovascular benefit
CV risk factors (BP, lipids, weight)	Significant reductions in SBP, triglycerides, weight, and waist circumference	Moderate improvements vs. placebo; less than tirzepatide	Tirzepatide shows stronger cardiometabolic risk-factor improvement
HF-related outcomes	No dedicated HF trial yet (*post-hoc* + RWE suggest benefit)	**STEP-HFpEF:** improved symptoms, function, inflammation	Semaglutide has dedicated HFpEF evidence

## Discussion

This narrative review synthesizes contemporary evidence comparing tirzepatide with semaglutide across glycemic, weight-related, and cardiometabolic domains. In completed randomized trials, tirzepatide consistently achieves greater reductions in body weight and HbA1c compared with semaglutide, demonstrating the synergistic metabolic benefits of simultaneous GIP and GLP-1 receptor stimulation.

### Cardiovascular outcomes: direct vs. contextual evidence

While metabolic outcomes strongly favor tirzepatide, the cardiovascular evidence base requires nuanced interpretation. Semaglutide possesses a well-established cardioprotective profile, supported by landmark trials (SUSTAIN-6, PIONEER-6, and SELECT) that demonstrate significant reductions in MACE (2, 9–13).

For tirzepatide, the SURPASS-CVOT trial provides robust contextual cardiovascular outcome data. Tirzepatide achieved non-inferiority to dulaglutide for three-point MACE in adults with T2DM, alongside superior improvements in glycemic control, body weight, blood pressure, and lipid parameters. Although superiority for the primary endpoint was not demonstrated against dulaglutide, the consistently favorable cardiometabolic risk-factor shifts suggest a potentially favorable cardiovascular profile (8).

### Critical appraisal of real-world evidence

Real-world observational studies supplement clinical trial data but reveal highly population-dependent differences. Some large analyses report lower rates of myocardial infarction, stroke, and all-cause mortality with tirzepatide compared with GLP-1 receptor agonists, including semaglutide (14, 15). Conversely, a target-trial emulation found small and variable cardiovascular differences between the two agents, though both outperformed non-incretin therapies (16). It is imperative to interpret these observational findings cautiously; disparities in outcomes may be heavily influenced by residual confounding factors, treatment selection bias, titration patterns, and heterogeneity in comparator definitions. Additionally, certain real-world findings currently originate from conference abstracts rather than fully peer-reviewed literature (17–20).

### Methodological limitations of comparative data

Several methodological limitations complicate direct comparisons between these agents. For instance, numerous semaglutide trials in diabetic cohorts utilized the 1.0 mg dosage rather than the 2.4 mg dosage approved for obesity, inherently skewing relative efficacy assessments (21, 22). Furthermore, trials exhibited significant variations in duration, intensity of lifestyle interventions, and background pharmacotherapy (4–7, 22, 23). Older subjects, non-White ethnic groups, and those with severe comorbidities are under-represented, which may limit the generalizability of the findings to real-world populations (24).

Both therapies exhibit favorable and comparable safety profiles, primarily characterized by transient gastrointestinal adverse events (4–7, 22, 25). However, discontinuation in real-world settings and submaximal titration rates significantly influence both real-world tolerability and sustained clinical effectiveness (26–28). Furthermore, recent withdrawal studies reveal significant weight regain following the cessation of therapy, highlighting the necessity for research focused on long-term maintenance strategies (25, 29).

## Conclusion

Tirzepatide and semaglutide represent highly effective pharmacologic options for managing obesity and T2DM, with distinct clinical evidence profiles. Based on direct head-to-head clinical trials, tirzepatide consistently achieves superior metabolic efficacy, specifically regarding weight loss and HbA1c reduction. Conversely, supported by independent, dedicated outcome trials, semaglutide currently retains the most mature and robust evidence for cardiovascular risk reduction. While contextual data confirms cardiovascular safety for tirzepatide against dulaglutide, definitive head-to-head cardiovascular outcomes comparing the two agents directly remain unestablished. Therefore, clinical selection must be highly individualized: tirzepatide should be prioritized for patients requiring maximal weight and glycemic improvements, whereas semaglutide is currently optimal for those requiring proven cardiovascular event reduction. Future research must prioritize definitive comparative cardiovascular outcome trials and rigorous real-world analyses to address current observational limitations and fully elucidate long-term comparative effectiveness.

## Data Availability

Publicly available datasets were analyzed in this study. This data can be found here: Clinicaltrials.gov.

## References

[1] Huang X Wu Y Ni Y Xu H He Y. Global, regional, and national burden of type 2 diabetes mellitus caused by high BMI from 1990 to 2021, and forecasts to 2045: analysis from the global burden of disease study 2021. Front Public Health. (2025) 13–2025. doi: 10.3389/fpubh.2025.1515797 39916706 PMC11798972

[2] Marso SP Bain SC Consoli A Eliaschewitz FG Jódar E Leiter LA . Semaglutide and cardiovascular outcomes in patients with type 2 diabetes. N Engl J Med. (2016) 375:1834–44. doi: 10.1056/NEJMoa1607141 27633186

[3] Marso SP Daniels GH Brown-Frandsen K Kristensen P Mann JFE Nauck MA . Liraglutide and cardiovascular outcomes in type 2 diabetes. N Engl J Med. (2016) 375:311–22. doi: 10.1056/NEJMoa1603827 27295427 PMC4985288

[4] Sorli C Harashima SI Tsoukas GM Unger J Karsbøl JD Hansen T . Efficacy and safety of once-weekly semaglutide monotherapy versus placebo in patients with type 2 diabetes (SUSTAIN 1): a double-blind, randomised, placebo-controlled, parallel-group, multinational, multicentre phase 3a trial. Lancet Diabetes Endocrinol. (2017) 5:251–60. doi: 10.1016/S2213-8587(17)30013-X 28110911

[5] Wilding JPH Batterham RL Calanna S Davies M Van Gaal LF Lingvay I . Once-weekly semaglutide in adults with overweight or obesity. N Engl J Med. (2021) 384:989–1002. doi: 10.1056/NEJMoa2032183 33567185

[6] Dahl D Onishi Y Norwood P Huh R Bray R Patel H . Effect of Subcutaneous tirzepatide vs placebo added to titrated insulin glargine on glycemic control in patients with type 2 diabetes: the SURPASS-5 randomized clinical trial. Jama. (2022) 327:534–45. doi: 10.1001/jama.2022.0078 35133415 PMC8826179

[7] Del Prato S Kahn SE Pavo I Weerakkody GJ Yang Z Doupis J . Tirzepatide versus insulin glargine in type 2 diabetes and increased cardiovascular risk (SURPASS-4): a randomised, open-label, parallel-group, multicentre, phase 3 trial. Lancet. (2021) 398:1811–24. doi: 10.1055/s-0042-1746267 34672967

[8] Nicholls SJ Bhatt DL Buse JB Prato SD Kahn SE Lincoff AM . Comparison of tirzepatide and dulaglutide on major adverse cardiovascular events in participants with type 2 diabetes and atherosclerotic cardiovascular disease: SURPASS-CVOT design and baseline characteristics. Am Heart J. (2024) 267:1–11. doi: 10.1016/j.ahj.2023.09.007 37758044

[9] Husain M Birkenfeld AL Donsmark M Dungan K Eliaschewitz FG Franco DR . Oral semaglutide and cardiovascular outcomes in patients with type 2 diabetes. N Engl J Med. (2019) 381:841–51. doi: 10.1056/NEJMoa1901118 31185157

[10] Lincoff AM Brown-Frandsen K Colhoun Helen M Deanfield J Emerson Scott S Esbjerg S . Semaglutide and cardiovascular outcomes in obesity without diabetes. N Engl J Med. (2023) 389:2221–32. doi: 10.1056/NEJMoa2307563 37952131

[11] Deanfield J Verma S Scirica BM Kahn SE Emerson SS Ryan D . Semaglutide and cardiovascular outcomes in patients with obesity and prevalent heart failure: a prespecified analysis of the SELECT trial. Lancet. (2024) 404:773–86. doi: 10.1016/S0140-6736(24)01498-3 39181597

[12] Kosiborod Mikhail N Abildstrøm Steen Z Borlaug Barry A Butler J Rasmussen S Davies M . Semaglutide in patients with heart failure with preserved ejection fraction and obesity. N Engl J Med. (2023) 389:1069–84. doi: 10.1056/NEJMoa2306963 37622681

[13] Kosiborod MN Verma S Borlaug BA Butler J Davies MJ Jon Jensen T . Effects of semaglutide on symptoms, function, and quality of life in patients with heart failure with preserved ejection fraction and obesity: a prespecified analysis of the STEP-HFpEF trial. Circulation. (2024) 149:204–16. doi: 10.1161/CIRCULATIONAHA.123.067505 37952180 PMC10782938

[14] Dani SS Makwana B Khadke S Kumar A Jhund P Nasir K . An observational study of cardiovascular outcomes of tirzepatide vs glucagon-like peptide-1 receptor agonists. JACC Adv. (2025) 4:101740. doi: 10.1016/j.jacadv.2025.101740 40447342 PMC12235410

[15] Tirzepatide Linked to Better CV Outcomes Than Semaglutide. Available online at: https://consultqd.clevelandclinic.org/tirzepatide-linked-to-better-heart-outcomes-than-semaglutide-in-masld-obesity-and-diabetes (Accessed July 10, 2025).

[16] Krüger N Schneeweiss S Desai RJ Sreedhara SK Kehoe AR Fuse K . Cardiovascular outcomes of semaglutide and tirzepatide for patients with type 2 diabetes in clinical practice. Nat Med. (2025) 32:342–52. doi: 10.1038/s41591-025-04102-x 41207920 PMC12823426

[17] Sherman RE Anderson SA Dal Pan GJ Gray GW Gross T Hunter NL . Real-world evidence - what is it and what can it tell us? N Engl J Med. (2016) 375:2293–7. doi: 10.1056/NEJMsb1609216 27959688

[18] Frieden TR. Evidence for health decision making - beyond randomized, controlled trials. N Engl J Med. (2017) 377:465–75. doi: 10.1056/NEJMra1614394 28767357

[19] Makady A de Boer A Hillege H Klungel O Goettsch W. What is real-world data? A review of definitions based on literature and stakeholder interviews. Value Health. (2017) 20:858–65. doi: 10.1016/j.jval.2017.03.008 28712614

[20] Osonoi T Oura T Hirase T. Glycaemic control, body weight, and safety of tirzepatide versus dulaglutide by baseline glycated haemoglobin level in Japanese patients with type 2 diabetes: a subgroup analysis of the SURPASS J-mono study. Diabetes Obes Metab. (2024) 26:126–34. doi: 10.1111/dom.15296 37794628

[21] Sinha R Papamargaritis D Sargeant JA Davies MJ. Efficacy and safety of tirzepatide in type 2 diabetes and obesity management. J Obes Metab Syndr. (2023) 32:25–45. doi: 10.7570/jomes22067 36750526 PMC10088547

[22] Davies M Færch L Jeppesen OK Pakseresht A Pedersen SD Perreault L. et al. Semaglutide 24 mg once a week in adults with overweight or obesity, and type 2 diabetes (STEP 2): a randomised, double-blind, double-dummy, placebo-controlled, phase 3 trial. Lancet. (2021) 397:971–84. doi: 10.1016/S0140-6736(21)00213-0 33667417

[23] Min T Bain SC. The role of tirzepatide, dual GIP and GLP-1 Receptor agonist, in the management of type 2 diabetes: the SURPASS clinical trials. Diabetes Ther. (2021) 12:143–57. doi: 10.1007/s13300-020-00981-0 33325008 PMC7843845

[24] Jeyakumar Y Richardson L Sarma S Retnakaran R Kramer CK. Representation of racialised and ethnically diverse populations in multicentre randomised controlled trials of GLP-1 medicines for obesity: a systematic review and meta-analysis of gaps. BMJ Glob Health. (2024) 9:e017177. doi: 10.1136/bmjgh-2024-017177 39608857 PMC11603712

[25] Rubino D Abrahamsson N Davies M Hesse D Greenway FL Jensen C . Effect of continued weekly subcutaneous semaglutide vs placebo on weight loss maintenance in adults with overweight or obesity: the STEP 4 randomized clinical trial. JAMA. (2021) 325:1414–25. doi: 10.1001/jama.2021.3224 33755728 PMC7988425

[26] Terrell K Vallarino CR Lozada JM Grabner M Teng CC Hoog MM . Real-world effectiveness of tirzepatide vs. semaglutide on HbA1c and weight in GLP-1 RA Naïve patients with T2D. ISPOR 2025 Meeting; May 13–16. (2025). Montréal, Québec, Canada. Value in Health. (2025). doi: 10.1016/j.jval.2025.04.108

[27] Trinh H Donovan A McAdam-Marx C. Real-world effectiveness of tirzepatide versus semaglutide for weight loss in overweight or obese patients in an ambulatory care setting. Diabetes Obes Metab. (2025) 27:3523–5. doi: 10.1111/dom.16343 40116184 PMC12046463

[28] Gasoyan H Butsch WS Schulte R Casacchia NJ Le P Boyer CB . Changes in weight and glycemic control following obesity treatment with semaglutide or tirzepatide by discontinuation status. Obesity. (2025) 33:1657–67. doi: 10.1002/oby.24331 40491239 PMC12381620

[29] Aronne LJ Horn DB. le Roux CW, Ho W, Falcon BL, Gomez Valderas E, et al. Tirzepatide as compared with semaglutide for the treatment of obesity. N Engl J Med. (2025) 393:26–36. doi: 10.1056/NEJMoa2416394 40353578

[30] Rodriguez PJ Goodwin Cartwright BM Gratzl S Brar R Baker C Gluckman TJ . Semaglutide vs tirzepatide for weight loss in adults with overweight or obesity. JAMA Intern Med. (2024) 184:1056–64. doi: 10.1001/jamainternmed.2024.2525 38976257 PMC11231910

[31] Chuang MH Chen JY Wang HY Jiang ZH Wu VC. Clinical outcomes of tirzepatide or GLP-1 receptor agonists in individuals with type 2 diabetes. JAMA Netw Open. (2024) 7:e2427258. doi: 10.1001/jamanetworkopen.2024.27258 39133485 PMC11320168

